# Methyl 4-(2,7-dimeth­oxy-1-naphtho­yl)benzoate

**DOI:** 10.1107/S160053681000382X

**Published:** 2010-02-06

**Authors:** Daichi Hijikata, Kosuke Nakaema, Shoji Watanabe, Akiko Okamoto, Noriyuki Yonezawa

**Affiliations:** a Department of Organic and Polymer Materials Chemistry, Tokyo University of Agriculture & Technology, Koganei, Tokyo 184-8588, Japan

## Abstract

In the title compound, C_21_H_18_O_5_, the dihedral angle between the naphthalene ring system and the benzene ring is 86.65 (6)°. The bridging carbonyl C—C(=O)—C plane makes dihedral angles of 83.57 (7) and 20.21 (8)°, respectively, with the naphthalene ring system and the benzene ring. The ester O—C=O plane and the benzene ring are almost coplanar, making a dihedral angle of 3.81 (18)°. The two meth­oxy groups lie essentially in the naphthalene ring plane [C—O—C—C torsion angles = 2.1 (2) and −1.44 (19)°]. In the crystal structure, a centrosymmetric dimer is formed through C—H⋯O bonds connecting the 7-meth­oxy group and the carbonyl O atom of the ester group. The dimers are further linked by C—H⋯O hydrogen bonds between the methoxy­carbonyl group and the bridging carbonyl O atom.

## Related literature

For electrophilic aromatic substitution of naphthalene derivatives, see: Okamoto & Yonezawa (2009 ▶). For the structures of closely related compounds, see: Mitsui, Nakaema, Noguchi *et al.* (2008 ▶); Mitsui, Nakaema, Noguchi & Yonezawa (2008 ▶); Mitsui *et al.* (2009 ▶); Watanabe *et al.* (2010 ▶).
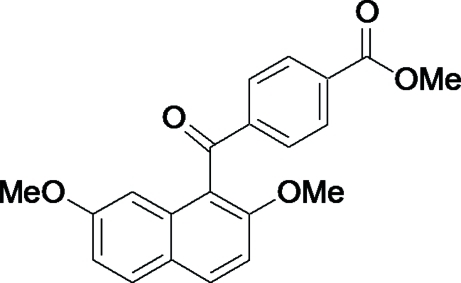


## Experimental

### 

#### Crystal data

C_21_H_18_O_5_
*M* *_r_* = 350.35
Triclinic, 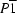

*a* = 7.7714 (2) Å
*b* = 9.5195 (3) Å
*c* = 12.2737 (4) Å
α = 97.525 (2)°
β = 97.919 (2)°
γ = 106.630 (2)°
*V* = 847.73 (5) Å^3^
*Z* = 2
Cu *K*α radiation
μ = 0.81 mm^−1^
*T* = 193 K
0.40 × 0.20 × 0.05 mm

#### Data collection

Rigaku R-AXIS RAPID diffractometer
Absorption correction: numerical (*NUMABS*; Higashi, 1999 ▶) *T*_min_ = 0.816, *T*_max_ = 0.960
15482 measured reflections
3066 independent reflections
2571 reflections with *I* > 2σ(*I*)
*R*_int_ = 0.029

#### Refinement

*R*[*F* ^2^ > 2σ(*F* ^2^)] = 0.037
*wR*(*F* ^2^) = 0.117
*S* = 1.08
3066 reflections
239 parameters
H-atom parameters constrained
Δρ_max_ = 0.24 e Å^−3^
Δρ_min_ = −0.20 e Å^−3^

### 

Data collection: *PROCESS-AUTO* (Rigaku, 1998 ▶); cell refinement: *PROCESS-AUTO*; data reduction: *CrystalStructure* (Rigaku/MSC, 2004 ▶); program(s) used to solve structure: *SIR2004* (Burla *et al.*, 2005 ▶); program(s) used to refine structure: *SHELXL97* (Sheldrick, 2008 ▶); molecular graphics: *ORTEPIII* (Burnett & Johnson, 1996 ▶); software used to prepare material for publication: *SHELXL97*.

## Supplementary Material

Crystal structure: contains datablocks global, I. DOI: 10.1107/S160053681000382X/is2518sup1.cif

Structure factors: contains datablocks I. DOI: 10.1107/S160053681000382X/is2518Isup2.hkl

Additional supplementary materials:  crystallographic information; 3D view; checkCIF report

## Figures and Tables

**Table 1:** Hydrogen-bond geometry (Å, °)

*D*—H⋯*A*	*D*—H	H⋯*A*	*D*⋯*A*	*D*—H⋯*A*
C19—H19*B*⋯O2^i^	0.98	2.57	3.461 (2)	152
C21—H21*A*⋯O1^ii^	0.98	2.49	3.4446 (19)	163

Symmetry codes: (i) 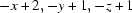; (ii) 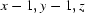.

**Fig. 1.**
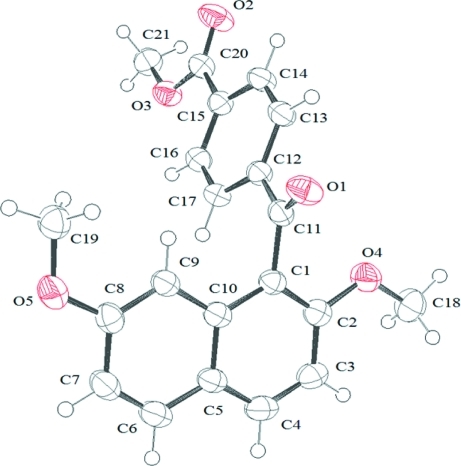
The molecular structure of (I), showing the atom-labeling scheme and 50% probability displacement ellipsoids.

**Fig. 2.**
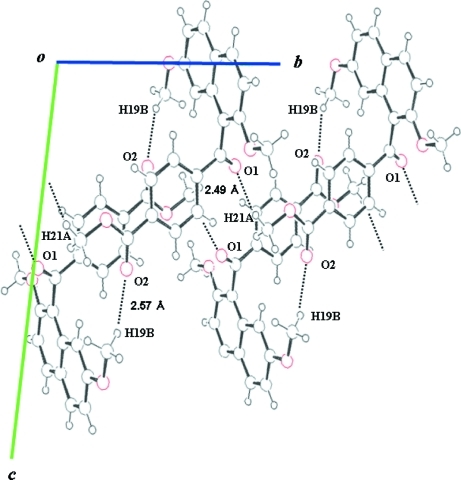
A partial crystal packing diagram of compound (I). The intermolecular C—H···O interactions are shown as dashed lines.

## supplementary crystallographic information

### Comment

In the course of our study on selective electrophilic aromatic aroylation of 2,7-dimethoxynaphthalene, *peri-*aroylnaphthalene compounds have proved to be formed regioselectively with the aid of suitable acidic mediator (Okamoto & Yonezawa, 2009). The aroyl groups at 1,8-positions of the naphthalene rings in these compounds are twisted almost perpendicularly but a little tiltedly toward *exo* sides against the naphthalene ring.

Recently, we have reported the X-ray crystal structures of several 1,8-diaroylated naphthalene homologues exemplified by bis(4-bromobenzoyl)(2,7-dimethoxynaphthalene-1,8-diyl)dimethanone (Watanabe *et al.*, 2010). Furthermore, we have also clarified the crystal structures of 1-monoaroylated naphthalenes. 1-(4-Chlorobenzoyl)-2,7-dimethoxynaphthalene (Mitsui, Nakaema, Noguchi, Okamoto & Yonezawa, 2008) and (4-chlorobenzoyl)(2-ethoxy-7-methoxynaphthalen-1-yl)methanone (Mitsui *et al.*, 2009) have essentially same non-coplanar structure as 1,8-diaroylated naphthalenes, and (4-chlorophenyl)(2-hydroxy-7-methoxynaphthalen-1-yl)methanone has substantially coplanar structure by intramolecular hydrogen bonding (Mitsui, Nakaema, Noguchi & Yonezawa, 2008). As a part of the course of our continuous study on the molecular structures of this kind of homologous molecules, the X-ray crystal structure of title compound, 1-monoaroylnaphthalene bearing ester group, is discussed in this report.

An *ORTEPIII *(Burnett & Johnson, 1996) plot of (I) is displayed in Fig. 1. In the molecule of (I), the interplanar angle between the benzene ring (C12—C17) and the naphthalene ring (C1—C10) is 86.65 (6)°. The dihedral angle between the ketonic C=O plane and the naphthalene ring is 83.57 (7)° [C10—C1—C11—O1 torsion angle = 84.41 (18)°]. The dihedral angle between the ketonic C=O plane and the benzene ring is 20.21 (8)° [C17—C12—C11—O1 torsion angle = 19.9 (2)°]. The torsion angle between the ketonic carbonyl group and benzene ring [C17—C12—C11—O1 torsion angle = 19.9 (2)°]  is larger than that between the carbonyl moiety of ester group and the benzene ring [C14—C15—C20—O2 torsion angle = -4.0 (2)°]. Two methoxy groups lie essentially on the naphthalene ring plane. The methyl group on O4, which is a part of methoxy group adjacent to the aroyl group, is oriented to the *exo* site of the molecule and that on O5 is directed to *endo* site. In the crystal packing, molecules are aligned forming dimeric pairs. Each pair has two intermolecular C—H···O bonds therein: equivalent hydrogen bonds between a H atom of 7-methoxy group (H19B) and the O atom of carbonyl moiety in ester group (O2). There is another type of hydrogen bond between dimeric pairs: hydrogen bonds between a H atom of the methyl moiety in ester group (H21A) and the O atom of ketonic carbonyl group (O1) (Fig. 2 and Table 1).

### Experimental

The title compound was prepared by regioselective electrophilic aromatic aroylation of 2,7-dimethoxynaphthalene with 4-(bromomethyl)benzoyl chloride followed by transformation of bromomethyl group. Single crystals suitable for X-ray diffraction were obtained by recrystallization from ethanol.

Spectroscopic Data: ^1^H NMR (300 MHz, CDCl_3_): *δ*3.73 (3*H*, s), 3.76 (3*H*, s), 3.94 (3*H*, s), 6.83 (1*H*, s), 7.01 (1*H*, d, *J *= 9.0 Hz), 7.14 (1*H*, d, *J* = 9.0 Hz), 7.71 (1*H*, d, *J* = 9.0 Hz), 7.87–7.90 (3*H*, m), 8.07 (2*H*, d, *J* = 8.1 Hz); ^13^C NMR (75.0 MHz, CDCl_3_): *δ*52.4, 55.2, 56.2, 101.9, 110.1, 117.2, 121.0, 124.4, 129.2, 129.7, 129.8, 131.6, 133.0, 133.9, 141.6, 155.4, 159.1, 166.4, 197.5; IR (KBr): 1674, 1625, 1511; m.p. = 154.6–157.1 °C; Anal. Calcd for C_21_H_18_O_5_: C 71.99, H 5.18%. Found: C 72.05, H 5.25%.

### Refinement

All H atoms were found in a difference map and were subsequently refined as riding atoms, with C—H = 0.95 (aromatic) and 0.98 (methyl) Å, and with *U*_iso_(H) = 1.2*U*_eq_(C).

### Crystal data

**Table:**

C_21_H_18_O_5_	*Z* = 2
*M**_r_* = 350.35	*F*(000) = 368
Triclinic, *P*1	*D*_x_ = 1.373 Mg m^−^^3^
Hall symbol: -P 1	Melting point = 427.6–430.1 K
*a* = 7.7714 (2) Å	Cu *K*α radiation, λ = 1.54187 Å
*b* = 9.5195 (3) Å	Cell parameters from 10631 reflections
*c* = 12.2737 (4) Å	θ = 3.7–68.2°
α = 97.525 (2)°	µ = 0.81 mm^−^^1^
β = 97.919 (2)°	*T* = 193 K
γ = 106.630 (2)°	Block, yellow
*V* = 847.73 (5) Å^3^	0.40 × 0.20 × 0.05 mm

### Data collection

**Table:**

Rigaku R-AXIS RAPID diffractometer	3066 independent reflections
Radiation source: rotating anode	2571 reflections with *I* > 2σ(*I*)
graphite	*R*_int_ = 0.029
Detector resolution: 10.00 pixels mm^-1^	θ_max_ = 68.2°, θ_min_ = 3.7°
ω scans	*h* = −9→9
Absorption correction: numerical (*NUMABS*; Higashi, 1999)	*k* = −11→11
*T*_min_ = 0.816, *T*_max_ = 0.960	*l* = −14→14
15482 measured reflections	

### Refinement

**Table:**

Refinement on *F*^2^	Secondary atom site location: difference Fourier map
Least-squares matrix: full	Hydrogen site location: difference Fourier map
*R*[*F*^2^ > 2σ(*F*^2^)] = 0.037	H-atom parameters constrained
*wR*(*F*^2^) = 0.117	*w* = 1/[σ^2^(*F*_o_^2^) + (0.0674*P*)^2^ + 0.1365*P*] where *P* = (*F*_o_^2^ + 2*F*_c_^2^)/3
*S* = 1.08	(Δ/σ)_max_ < 0.001
3066 reflections	Δρ_max_ = 0.24 e Å^−^^3^
239 parameters	Δρ_min_ = −0.20 e Å^−^^3^
0 restraints	Extinction correction: *SHELXL97* (Sheldrick, 2008), Fc^*^=kFc[1+0.001xFc^2^λ^3^/sin(2θ)]^-1/4^
Primary atom site location: structure-invariant direct methods	Extinction coefficient: 0.0056 (9)

### Special details

**Table:**

Geometry. All e.s.d.'s (except the e.s.d. in the dihedral angle between two l.s. planes) are estimated using the full covariance matrix. The cell e.s.d.'s are taken into account individually in the estimation of e.s.d.'s in distances, angles and torsion angles; correlations between e.s.d.'s in cell parameters are only used when they are defined by crystal symmetry. An approximate (isotropic) treatment of cell e.s.d.'s is used for estimating e.s.d.'s involving l.s. planes.
Refinement. Refinement of *F*^2^ against ALL reflections. The weighted *R*-factor *wR* and goodness of fit *S* are based on *F*^2^, conventional *R*-factors *R* are based on *F*, with *F* set to zero for negative *F*^2^. The threshold expression of *F*^2^ > σ(*F*^2^) is used only for calculating *R*-factors(gt) *etc*. and is not relevant to the choice of reflections for refinement. *R*-factors based on *F*^2^ are statistically about twice as large as those based on *F*, and *R*- factors based on ALL data will be even larger.

### Fractional atomic coordinates and isotropic or equivalent isotropic displacement parameters (Å^2^)

**Table:**

	*x*	*y*	*z*	*U*_iso_*/*U*_eq_	
O1	1.18952 (13)	0.98013 (12)	0.33288 (8)	0.0454 (3)	
O2	0.76893 (17)	0.46407 (13)	0.65183 (9)	0.0570 (3)	
O3	0.59054 (13)	0.32936 (11)	0.49174 (8)	0.0423 (3)	
O4	0.76938 (14)	1.00486 (13)	0.26247 (8)	0.0470 (3)	
O5	1.29922 (14)	0.59184 (12)	0.00036 (9)	0.0479 (3)	
C1	0.94439 (17)	0.87694 (14)	0.17801 (11)	0.0320 (3)	
C2	0.80587 (18)	0.93926 (15)	0.16515 (12)	0.0363 (3)	
C3	0.7095 (2)	0.93512 (17)	0.05840 (12)	0.0420 (4)	
H3	0.6144	0.9794	0.0502	0.050*	
C4	0.7548 (2)	0.86624 (17)	−0.03362 (12)	0.0431 (4)	
H4	0.6898	0.8635	−0.1058	0.052*	
C5	0.89479 (18)	0.79937 (15)	−0.02438 (11)	0.0351 (3)	
C6	0.9413 (2)	0.72579 (16)	−0.11830 (12)	0.0426 (4)	
H6	0.8780	0.7228	−0.1910	0.051*	
C7	1.0735 (2)	0.65969 (16)	−0.10717 (12)	0.0439 (4)	
H7	1.1012	0.6098	−0.1715	0.053*	
C8	1.17077 (19)	0.66469 (15)	0.00021 (12)	0.0375 (3)	
C9	1.13305 (18)	0.73542 (15)	0.09430 (11)	0.0342 (3)	
H9	1.1997	0.7383	0.1659	0.041*	
C10	0.99256 (17)	0.80497 (14)	0.08386 (11)	0.0326 (3)	
C11	1.04313 (18)	0.88600 (15)	0.29455 (11)	0.0334 (3)	
C12	0.95508 (17)	0.77299 (15)	0.36010 (11)	0.0328 (3)	
C13	1.00721 (19)	0.79937 (16)	0.47627 (11)	0.0370 (3)	
H13	1.0970	0.8899	0.5134	0.044*	
C14	0.92869 (19)	0.69435 (16)	0.53730 (11)	0.0391 (3)	
H14	0.9634	0.7133	0.6164	0.047*	
C15	0.79879 (18)	0.56076 (16)	0.48323 (11)	0.0352 (3)	
C16	0.74621 (19)	0.53386 (16)	0.36727 (11)	0.0370 (3)	
H16	0.6573	0.4429	0.3301	0.044*	
C17	0.82356 (18)	0.63965 (15)	0.30640 (11)	0.0358 (3)	
H17	0.7869	0.6214	0.2274	0.043*	
C18	0.6225 (2)	1.0673 (2)	0.25460 (15)	0.0519 (4)	
H18A	0.6111	1.1086	0.3297	0.062*	
H18B	0.5085	0.9895	0.2185	0.062*	
H18C	0.6472	1.1467	0.2101	0.062*	
C19	1.4033 (2)	0.59325 (18)	0.10659 (13)	0.0460 (4)	
H19A	1.4865	0.5344	0.0961	0.055*	
H19B	1.3203	0.5502	0.1553	0.055*	
H19C	1.4742	0.6961	0.1411	0.055*	
C20	0.72025 (19)	0.44915 (16)	0.55271 (11)	0.0381 (3)	
C21	0.5110 (2)	0.21403 (17)	0.55273 (13)	0.0466 (4)	
H21A	0.4122	0.1347	0.5020	0.056*	
H21B	0.4621	0.2568	0.6138	0.056*	
H21C	0.6051	0.1726	0.5835	0.056*	

### Atomic displacement parameters (Å^2^)

**Table:**

	*U*^11^	*U*^22^	*U*^33^	*U*^12^	*U*^13^	*U*^23^
O1	0.0398 (6)	0.0492 (6)	0.0360 (5)	0.0012 (5)	0.0004 (4)	0.0041 (5)
O2	0.0752 (8)	0.0539 (7)	0.0302 (6)	0.0028 (6)	0.0075 (5)	0.0079 (5)
O3	0.0465 (6)	0.0410 (6)	0.0352 (5)	0.0071 (4)	0.0065 (4)	0.0075 (4)
O4	0.0489 (6)	0.0594 (7)	0.0394 (6)	0.0285 (5)	0.0095 (5)	0.0043 (5)
O5	0.0547 (6)	0.0520 (6)	0.0406 (6)	0.0230 (5)	0.0140 (5)	0.0008 (5)
C1	0.0331 (7)	0.0312 (7)	0.0297 (7)	0.0068 (5)	0.0052 (5)	0.0061 (5)
C2	0.0357 (7)	0.0351 (7)	0.0363 (7)	0.0084 (6)	0.0066 (6)	0.0063 (6)
C3	0.0391 (8)	0.0460 (8)	0.0414 (8)	0.0157 (6)	0.0006 (6)	0.0116 (7)
C4	0.0450 (8)	0.0437 (8)	0.0349 (7)	0.0074 (6)	−0.0021 (6)	0.0113 (6)
C5	0.0393 (7)	0.0317 (7)	0.0285 (7)	0.0033 (6)	0.0031 (5)	0.0059 (5)
C6	0.0530 (9)	0.0391 (8)	0.0283 (7)	0.0047 (7)	0.0041 (6)	0.0054 (6)
C7	0.0572 (9)	0.0394 (8)	0.0313 (7)	0.0098 (7)	0.0117 (6)	0.0017 (6)
C8	0.0407 (7)	0.0325 (7)	0.0374 (7)	0.0077 (6)	0.0112 (6)	0.0035 (6)
C9	0.0383 (7)	0.0328 (7)	0.0292 (7)	0.0077 (6)	0.0065 (5)	0.0048 (5)
C10	0.0355 (7)	0.0289 (7)	0.0290 (7)	0.0030 (5)	0.0055 (5)	0.0058 (5)
C11	0.0361 (7)	0.0349 (7)	0.0290 (7)	0.0130 (6)	0.0059 (5)	0.0006 (5)
C12	0.0338 (7)	0.0366 (7)	0.0287 (7)	0.0139 (6)	0.0047 (5)	0.0028 (5)
C13	0.0401 (7)	0.0375 (7)	0.0290 (7)	0.0096 (6)	0.0020 (5)	0.0005 (6)
C14	0.0452 (8)	0.0445 (8)	0.0251 (6)	0.0127 (6)	0.0039 (5)	0.0027 (6)
C15	0.0388 (7)	0.0392 (8)	0.0304 (7)	0.0164 (6)	0.0073 (5)	0.0057 (6)
C16	0.0414 (7)	0.0345 (7)	0.0317 (7)	0.0099 (6)	0.0024 (6)	0.0023 (6)
C17	0.0414 (7)	0.0387 (8)	0.0254 (6)	0.0129 (6)	0.0020 (5)	0.0027 (6)
C18	0.0459 (9)	0.0587 (10)	0.0587 (10)	0.0274 (8)	0.0132 (7)	0.0078 (8)
C19	0.0449 (8)	0.0487 (9)	0.0463 (9)	0.0180 (7)	0.0103 (6)	0.0051 (7)
C20	0.0428 (8)	0.0407 (8)	0.0310 (7)	0.0140 (6)	0.0078 (6)	0.0041 (6)
C21	0.0498 (9)	0.0439 (9)	0.0462 (9)	0.0121 (7)	0.0118 (7)	0.0109 (7)

### Geometric parameters (Å, °)

**Table:**

O1—C11	1.2154 (16)	C9—C10	1.4298 (19)
O2—C20	1.2002 (17)	C9—H9	0.9500
O3—C20	1.3368 (17)	C11—C12	1.4933 (18)
O3—C21	1.4499 (17)	C12—C17	1.3939 (19)
O4—C2	1.3772 (17)	C12—C13	1.3960 (18)
O4—C18	1.4277 (18)	C13—C14	1.381 (2)
O5—C8	1.3687 (17)	C13—H13	0.9500
O5—C19	1.4327 (18)	C14—C15	1.391 (2)
C1—C2	1.3708 (19)	C14—H14	0.9500
C1—C10	1.4154 (19)	C15—C16	1.3937 (19)
C1—C11	1.5065 (18)	C15—C20	1.496 (2)
C2—C3	1.407 (2)	C16—C17	1.3813 (19)
C3—C4	1.370 (2)	C16—H16	0.9500
C3—H3	0.9500	C17—H17	0.9500
C4—C5	1.408 (2)	C18—H18A	0.9800
C4—H4	0.9500	C18—H18B	0.9800
C5—C6	1.413 (2)	C18—H18C	0.9800
C5—C10	1.4231 (18)	C19—H19A	0.9800
C6—C7	1.351 (2)	C19—H19B	0.9800
C6—H6	0.9500	C19—H19C	0.9800
C7—C8	1.414 (2)	C21—H21A	0.9800
C7—H7	0.9500	C21—H21B	0.9800
C8—C9	1.3695 (19)	C21—H21C	0.9800
			
C20—O3—C21	115.70 (11)	C13—C12—C11	119.85 (12)
C2—O4—C18	118.25 (12)	C14—C13—C12	120.21 (13)
C8—O5—C19	117.18 (11)	C14—C13—H13	119.9
C2—C1—C10	120.65 (12)	C12—C13—H13	119.9
C2—C1—C11	118.48 (12)	C13—C14—C15	120.17 (12)
C10—C1—C11	120.86 (12)	C13—C14—H14	119.9
C1—C2—O4	115.70 (12)	C15—C14—H14	119.9
C1—C2—C3	121.08 (14)	C14—C15—C16	119.85 (13)
O4—C2—C3	123.22 (13)	C14—C15—C20	118.23 (12)
C4—C3—C2	118.99 (14)	C16—C15—C20	121.91 (12)
C4—C3—H3	120.5	C17—C16—C15	119.91 (13)
C2—C3—H3	120.5	C17—C16—H16	120.0
C3—C4—C5	121.91 (13)	C15—C16—H16	120.0
C3—C4—H4	119.0	C16—C17—C12	120.43 (12)
C5—C4—H4	119.0	C16—C17—H17	119.8
C4—C5—C6	122.64 (13)	C12—C17—H17	119.8
C4—C5—C10	118.76 (13)	O4—C18—H18A	109.5
C6—C5—C10	118.61 (13)	O4—C18—H18B	109.5
C7—C6—C5	121.56 (13)	H18A—C18—H18B	109.5
C7—C6—H6	119.2	O4—C18—H18C	109.5
C5—C6—H6	119.2	H18A—C18—H18C	109.5
C6—C7—C8	120.04 (14)	H18B—C18—H18C	109.5
C6—C7—H7	120.0	O5—C19—H19A	109.5
C8—C7—H7	120.0	O5—C19—H19B	109.5
O5—C8—C9	124.51 (13)	H19A—C19—H19B	109.5
O5—C8—C7	114.37 (12)	O5—C19—H19C	109.5
C9—C8—C7	121.10 (14)	H19A—C19—H19C	109.5
C8—C9—C10	119.48 (13)	H19B—C19—H19C	109.5
C8—C9—H9	120.3	O2—C20—O3	123.51 (13)
C10—C9—H9	120.3	O2—C20—C15	124.10 (13)
C1—C10—C5	118.60 (13)	O3—C20—C15	112.39 (11)
C1—C10—C9	122.18 (12)	O3—C21—H21A	109.5
C5—C10—C9	119.20 (12)	O3—C21—H21B	109.5
O1—C11—C12	121.49 (12)	H21A—C21—H21B	109.5
O1—C11—C1	121.30 (12)	O3—C21—H21C	109.5
C12—C11—C1	117.20 (11)	H21A—C21—H21C	109.5
C17—C12—C13	119.41 (12)	H21B—C21—H21C	109.5
C17—C12—C11	120.72 (11)		
			
C10—C1—C2—O4	179.30 (11)	C6—C5—C10—C9	−0.47 (18)
C11—C1—C2—O4	−0.32 (18)	C8—C9—C10—C1	178.58 (11)
C10—C1—C2—C3	−0.8 (2)	C8—C9—C10—C5	−0.08 (18)
C11—C1—C2—C3	179.60 (12)	C2—C1—C11—O1	−98.98 (16)
C18—O4—C2—C1	−177.96 (12)	C10—C1—C11—O1	81.40 (17)
C18—O4—C2—C3	2.1 (2)	C2—C1—C11—C12	82.03 (15)
C1—C2—C3—C4	0.6 (2)	C10—C1—C11—C12	−97.59 (14)
O4—C2—C3—C4	−179.50 (13)	O1—C11—C12—C17	−158.78 (14)
C2—C3—C4—C5	0.1 (2)	C1—C11—C12—C17	20.22 (18)
C3—C4—C5—C6	178.96 (13)	O1—C11—C12—C13	19.9 (2)
C3—C4—C5—C10	−0.5 (2)	C1—C11—C12—C13	−161.07 (12)
C4—C5—C6—C7	−178.55 (13)	C17—C12—C13—C14	−0.2 (2)
C10—C5—C6—C7	0.9 (2)	C11—C12—C13—C14	−178.91 (12)
C5—C6—C7—C8	−0.8 (2)	C12—C13—C14—C15	0.8 (2)
C19—O5—C8—C9	−1.44 (19)	C13—C14—C15—C16	−0.8 (2)
C19—O5—C8—C7	179.73 (12)	C13—C14—C15—C20	178.81 (13)
C6—C7—C8—O5	179.11 (12)	C14—C15—C16—C17	0.2 (2)
C6—C7—C8—C9	0.2 (2)	C20—C15—C16—C17	−179.44 (13)
O5—C8—C9—C10	−178.55 (11)	C15—C16—C17—C12	0.5 (2)
C7—C8—C9—C10	0.2 (2)	C13—C12—C17—C16	−0.5 (2)
C2—C1—C10—C5	0.31 (19)	C11—C12—C17—C16	178.25 (12)
C11—C1—C10—C5	179.92 (11)	C21—O3—C20—O2	−0.9 (2)
C2—C1—C10—C9	−178.35 (12)	C21—O3—C20—C15	178.40 (12)
C11—C1—C10—C9	1.26 (19)	C14—C15—C20—O2	−4.0 (2)
C4—C5—C10—C1	0.33 (18)	C16—C15—C20—O2	175.62 (15)
C6—C5—C10—C1	−179.18 (11)	C14—C15—C20—O3	176.73 (12)
C4—C5—C10—C9	179.04 (11)	C16—C15—C20—O3	−3.64 (19)

### Hydrogen-bond geometry (Å, °)

**Table:**

*D*—H···*A*	*D*—H	H···*A*	*D*···*A*	*D*—H···*A*
C19—H19B···O2^i^	0.98	2.57	3.461 (2)	152
C21—H21A···O1^ii^	0.98	2.49	3.4446 (19)	163

Symmetry codes: (i) −*x*+2, −*y*+1, −*z*+1; (ii) *x*−1, *y*−1, *z*.
